# A Very Rare Variant in *SREBF2*, a Possible Cause of Hypercholesterolemia and Increased Glycemic Levels

**DOI:** 10.3390/biomedicines10051178

**Published:** 2022-05-19

**Authors:** Ana-Bárbara García-García, Sergio Martínez-Hervás, Santiago Vernia, Carmen Ivorra, Inés Pulido, Juan-Carlos Martín-Escudero, Marta Casado, Julián Carretero, José T. Real, Felipe Javier Chaves

**Affiliations:** 1 CIBER of Diabetes and Associated Metabolic Diseases (CIBERDEM), 28029 Madrid, Spain; a.barbara.garcia@ext.uv.es (A.-B.G.-G.); sergio.martinez@uv.es (S.M.-H.); jose.t.real@uv.es (J.T.R.); 2 Genomic and Diabetes Unit, INCLIVA Biomedical Research Institute, 46010 Valencia, Spain; ivorra@seqplexing.com; 3 Department of Medicine, University of Valencia, 46010 Valencia, Spain; 4 Endocrinology Service, University Clinical Hospital of Valencia, 46010 Valencia, Spain; 5 INCLIVA Biomedical Research Institute, 46010 Valencia, Spain; 6 Biomedical Institute of Valencia (IBV-CSIC), 46010 Valencia, Spain; santiago.vernia@lms.mrc.ac.uk (S.V.); mcasado@ibv.csic.es (M.C.); 7 University of Illinois Hospital & Health Sciences System Cancer Center, University of Illinois Chicago, Chicago, IL 60612, USA; ipulid3@uic.edu; 8 Department of Physiology, University of Valencia, 46010 Valencia, Spain; julian.carretero@uv.es; 9 Internal Medicine Service, Rio Hortega Hospital, 47012 Valladolid, Spain; juancarlos.martinescudero@uva.es; 10 CIBER of Hepatic and Digestive Diseases (CIBEREHD), 28029 Madrid, Spain

**Keywords:** hyperglycemia, hyperlipidemia, diabetes, genetic disease, SREBP system, mutation

## Abstract

Patients with high cholesterol and glucose levels are at high risk for cardiovascular disease. The Sterol Regulatory Element Binding Protein (SREBP) system regulates genes involved in lipid, cholesterol and glucose pathways. Autosomal Dominant Hypercholesterolemias (ADHs) are a group of diseases with increased cholesterol levels. They affect 1 out of every 500 individuals. About 20–30% of patients do not present any mutation in the known genes (*LDLR*, *APOB* and *PCSK9*). ADHs constitute a good model to identify the genes involved in the alteration of lipid levels or possible therapeutic targets. In this paper, we studied whether a mutation in the SREBP system could be responsible for ADH and other metabolic alterations present in these patients. Forty-one ADH patients without mutations in the main responsible genes were screened by direct sequencing of SREBP system genes. A luciferase reporter assay of the found mutation and an oral glucose tolerance test in carriers and non-carriers were performed. We found a novel mutation in the *SREBF2* gene that increases transcription levels and cosegregates with hypercholesterolemia, and we found increased glucose levels in one family. SREBP2 is known to be involved in cholesterol synthesis, plasma levels and glucose metabolism in humans. The found mutation may involve the *SREBF2* gene in hypercholesterolemia combined with hyperglycemia.

## 1. Introduction

Elevated levels of plasma low-density lipoprotein cholesterol (LDLc) and elevated levels of glucose are two of the main risk factors for coronary artery disease (CAD), one of the most frequent causes of death in Western society [1,2,3]. LDLc and glucose levels are multifactorial and can be modulated by different genes.

Sterol Regulatory Element Binding Proteins (SREBPs) regulate the transcription of common genes involved in cholesterol and fatty acid biosynthesis. SREBP1a and SREBP1c are encoded by *SREBF1* and primarily regulate genes involved in fatty acid, cholesterol, lipid and glucose metabolism. *SREBF2* is mainly involved in cholesterol and lipid homeostasis, in both the liver and adipose tissue [4,5]. Some proteins regulate SREBP activity: membrane-bound transcription factor peptidase sites 1 and 2 (MBTPS1 and MBTPS2, respectively), and INSIG1. Recently, SREBP2 has been found to be related to SREBP1c and SREBP1a regulation in the liver, implying that SREBP2 could modulate the function of the whole system [6]. In addition, the overexpression of SREBP2 can lead to an alteration in insulin secretion, a reduced number of pancreatic beta cells, diabetes, low weight, increased total cholesterol (TC) in blood and tissues, and non-alcoholic hepatic steatosis [7]. In humans, some studies associate *SREBF2* variants with hypercholesterolemia [8], insulin resistance, DM and liver steatosis [9].

Autosomal Dominant Hypercholesterolemias (ADHs, OMIM 143890) are a suitable model for the study of primary hypercholesterolemia. ADHs are a series of diseases characterized by increased plasma total and LDLc and increased cardiovascular risk [10]. ADH syndromes vary based on the gene harboring the causative defect. Mutations in the LDL receptor (*LDLR*), Apolipoprotein B (*APOB*) and Proprotein Convertase Subtilisin/Kexin type 9 (*PCSK9*) genes are the most commonly recognized loci [11], although in up to 20% of cases, the genetic defect is unknown [12]. Mutations may be consulted in the Human Gene Mutation Database (HGMD) [13]. Most ADH patients develop CAD [10]. In addition, Diabetes Mellitus type 2 is an independent CAD risk factor, and it has been shown that the cardiovascular disease mortality risk is higher in diabetic patients with increased TC levels [14].

The objective of this work was to study the presence of mutations in the SREBP system, which could be responsible for hypercholesterolemia in a group of patients with ADH without evidence of the most commonly associated mutations known to date.

## 2. Materials and Methods

### 2.1. Subjects

The genetic study was conducted in 41 subjects, without any mutation in the *LDLR*, *APOB*, or *PCSK9* genes, from an initial group of 150 ADH patients attending our Lipid Clinic. ADH was diagnosed following the Med Ped criteria [15]: plasma levels of total and LDLc higher than the 95th percentile, corrected for both age and sex, together with the presence of tendon xanthomas, CAD in the proband or in a first-degree relative, and a bimodal distribution of TC and LDLc levels in the family (autosomal dominant pattern of lipid IIa phenotype). A clinical examination was performed, and clinical history was obtained.

Mutations found in ADH patients were screened in two samples: 429 healthy controls and 1000 hyperglycemic and/or hypercholesterolemic subjects from the general population. DNA was obtained from the August Pi i Sunyer Biomedical Research Institute (IDIBAPS) biobank, which aims to provide the scientific community with high-quality samples for all types of pathologies. Due to the impossibility of obtaining OGTT data from these controls, 28 healthy subjects were randomly selected from a total of 260 subjects who were previously selected for studies of cut points for insulin resistance in the Spanish population [16,17]. Both 429 and 28 controls had TC and LDLc levels lower than the 70th percentile of our population, did not have glucose alterations and were not under any treatment capable of modifying lipid or glucose levels.

All patients gave their written consent. The Institutional Ethics Committee from the INCLIVA Biomedical Research Institute approved the work. We are indebted to the IDIBAPS Biobank for the above-mentioned samples and data procurement.

### 2.2. DNA Analysis

DNA was extracted from whole blood as described by Tilzer [18] using the Chemagic System (Chemagen, Pelkin Elmer, Baesweiler, Germany), following the manufacturer’s guidelines.

The screening for *LDLR* gene mutations was performed by looking for large rearrangements using Southern blot and a semiquantitative method designed by our group [19,20], and by looking for small mutations by sequencing all exons, their corresponding intron–exon junctions and about 500 base pairs of the promoter [21]. Exons 26 and 29 from the *APOB* gene, where ADH mutations have been found [22], were sequenced. The *PCSK9* gene was screened by direct sequencing of the promoter, exons and splice site regions [23]. 

All exons from the *SREBF2*, *SREBF1*, *MBTPS1*, *MBTPS2* and *INSIG1* genes, their splice site junctions and approximately 700 bp from the promoter regions were amplified and sequenced in an ABI 3730 system. The genetic sequences used as references are the following: *SREBF2*: NC_000022.9 (genomic sequence), NM_004599.2 (cDNA); *SREBF1*: NC_000017.9 (genomic), NM_004176.3 (cDNA); *MBTPS1*: NC_000016.8 (genomic), NM_003791.2 (cDNA); *MBTPS2*: NC_000023.9 (genomic), NM_015884.1 (cDNA); and *INSIG1*: NC_000007.12 (genomic), NM_198336.1 (cDNA). 

The genes known to cause the most frequent Maturity-Onset Diabetes of the Young (MODY) types (*GCK*, *HNF1A* and *HNF4A*) were analyzed by the direct sequencing of the promoter, exons and intron splice junctions (*GCK*: NC_000007.1 (genomic), NM_000162.2 (cDNA); *HNF1A*: NC_000012.10 (genomic), NM_000545.4 (cDNA); *HNF4A*: NC_000020.9 (genomic), NM_175914.3 (cDNA)).

All primers used to analyze the above genes were designed in our laboratory with the program PRIMER 3 [24] and may be consulted in the Supplementary Materials (Table S1).

### 2.3. Pgl3-Srebp2 Promoter–Reporter Gene Constructs 

SREBF2 promoter/luciferase reporter constructs were made by cloning an NcoI/NcoI-flanked insert, encoding the proximal portion of the wild type or c.-405 A>G SREBF2 promoter into the pGL3-Basic vector (Promega Biotech Iberica S.L, Alcobendas, Spain). The insert was obtained by PCR from the genomic DNA of a heterozygous c.-405A>G subject using sense and antisense primers. The integrity of each construct was verified by sequencing in both directions:

Sense primer: 5′-TGGAAGCTCTCAGAGGG-3′;

Antisense primer: 5′-CGCCGCTGTCGTCCATGGCCCGGCTCAGCGCAG-3′.

### 2.4. Cell Culture and Luciferase Assay

For the promoter assay, Hep G2, Caco-2 and 3T3-L1 cells were grown in DMEM with 25 mM glucose, supplemented with 10% fetal bovine serum (FBS) and antibiotics (100 U/mL penicillin and 100 µg/mL streptomycin) at 37 °C in a humidified atmosphere of 5% CO_2_. Transfection with the different plasmids was performed on attached cells at 70% confluence, using TransIT-LT1 Transfection Reagent (Mirus-Bio LLC, Madison, WI, USA) and employing 750 ng of pGL3-SREBF2 reporter constructs (wild type or mutated) and 40 ng of a promoterless Renilla luciferase construct (pRL-0). Transactivation activities were measured 24 h after transfection in a Wallac 1420 VICTOR luminometer using a Dual-Luciferase Reporter Assay System (Promega Biotech Iberica, Alcobendas, Spain), following the manufacturer´s instructions. Relative light units were determined by quantification of the signal from Firefly luciferase, normalized with co-transfected Renilla luciferase activity in the same sample. Finally, these relative values were normalized against mock transfection. Each expression construct was transfected in triplicate wells. The experiments were repeated three times. 

### 2.5. Oral Glucose Tolerance Test

An oral glucose tolerance test (OGTT) was carried out in the following individuals: four relatives heterozygous for the *SREBF2* c.-405A>G mutation and with a clinical diagnosis of ADH who were matched by Body Mass Index (BMI), age and gender with 27 controls. These controls were randomly selected from a total of 260 subjects from a group previously selected for studies of cut points for insulin resistance in our population [16,17]. 

A dose of 75g of glucose was given to participants, and blood samples were drawn at times of 0, 30, 60, 90 and 120 minutes. Glucose and insulin levels were determined at these points. Homeostasis Model Assessment (HOMA) calculations were performed as described [25].

### 2.6. Statistical Analysis

Values are given as mean ± SD (unless indicated otherwise). In cultured cell experiments, one-way ANOVA with Bonferroni correction or the T-test procedure was used to analyze differences between and within groups. A *p*-value < 0.05 was considered significant. A non-parametric test for two independent samples (Mann–Whitney U) was performed to compare data from the OGTT experiment between controls and carriers, who were matched by age, gender and BMI.

## 3. Results

### 3.1. Genetic Analysis

We screened the SREBP system for potential causal mutations, including the *SREBF1*, *SREBF2*, *MBTPS1*, *MBTPS2* and *INSIG1* genes, in 41 ADH patients without genetic defects in the *LDLR*, *ApoB* or *PCSK9* genes. 

We identified a c.-405A>G variant in the *SREBF2* gene, which could be expected to alter *SREBF2* functionality. This position is conserved in 7 out of 10 mammals analyzed (with available DNA sequence of this region) and, overall, in the five *Hominidae* species (orangutan, bonobo, chimpanzee, gorilla and human), suggesting that changes in this position are under negative selection (Supplementary Figure S1). We did not find any mutation expected to be pathogenic in the other studied genes.

**Figure 1:**
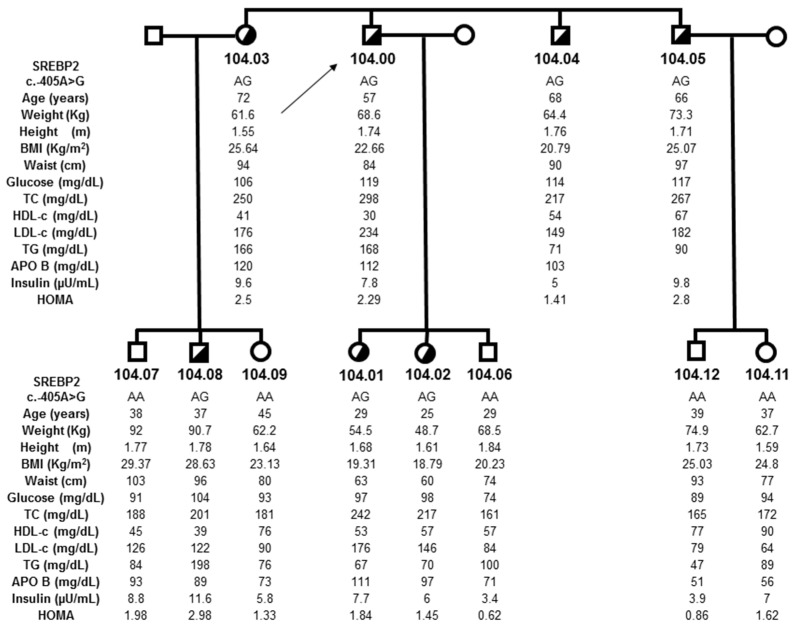
Pedigree structure and clinical data from family 104. Index case is identified with an arrow. ADH patients are indicated with half-filled symbols (squares: male individuals; circumferences: female individuals) (TG: triglycerides).

The potential functional role of the mutation in *SREBF2* was evaluated by screening a control sample and by studying cosegregation in the family. We did not find a c.-405G mutation in 429 normocholesterolemic and normoglycemic controls or in 1000 subjects from the general population who were hyperglycemic and/or hypercholesterolemic. In the carrier’s family, we found the mutation in seven co-sanguineous subjects, all with hypercholesterolemia, although cholesterol elevations were modest (Figure 1). 

### 3.2. SREBP2 Promoter Assays

**Figure 2:**
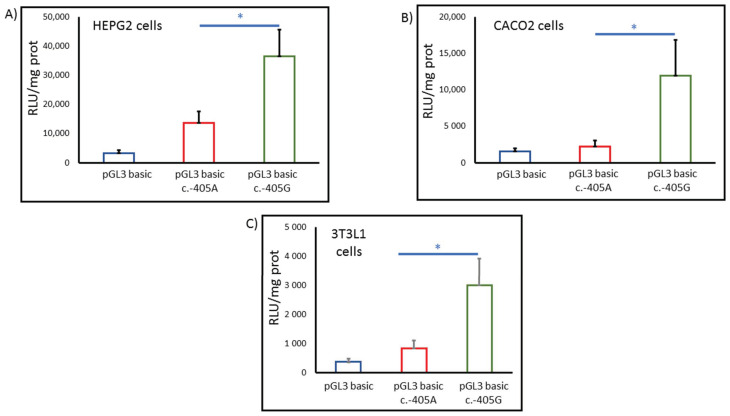
Transcription activity analysis of the -1094/-1 region of SREBF2 human promoter in several cell types ((**A**): HepG2 cells, (**B**): Caco2 cells, (**C**): 3T3L1 cells). Cells were transiently transfected with SREBF2 luciferase reporter constructs plus promoterless Renilla plasmid. Data represent mean ± SD and are expressed as fold induction with respect to the empty vector (pGL3basic) (* significant).

We further examined whether this variant could alter gene transcription by transfecting HepG2, Caco2 and 3T3L cell lines with wild-type plasmids and plasmids containing the mutation c.-405A>G, and by performing a luciferase reporter assay. Our results indicate that cells with the mutant construct exhibit higher transcription activity in different cell types (between 2.0 and 3.6 times) than the wild-type promoter (*p* < 0.05) (Figure 2).

### 3.3. Promoter Mutation and Glucose Levels

The carriers of variant c.-405G were also found to have elevations in plasma glucose levels (ranging from 97 to 119 mg/dL) (Figure 1). Proband 104.00 was hyperglycemic, and the remaining carriers were found to have fasting glucose levels in the prediabetic range. 

In order to exclude the presence of MODY, we analyzed the three genes most frequently responsible for MODY, namely, the *GCK*, *HNF1A* and *HNF4A* genes, and we did not find any mutation that could cause MODY. In order to exclude the presence of Latent Autoimmune Diabetes in Adults (LADA), we tested the presence of GAD+ autoantibodies and islet cell antibodies. We did not find these antibodies in any of the c-405G carriers, thereby ruling out the presence of LADA. 

### 3.4. Promoter Mutation and Cholesterol and Glucose Levels

Biochemical and anthropometric characteristics of carriers for the G allele, non-carrier relatives, and the 429 normocholesterolemic and normoglycemic controls screened for this variation were compared (Table 1). Carriers were found to have higher fasting glucose levels, TC and LDLC than both related non-carriers and unrelated 429 controls, but there were no significant differences between non-carrier relatives and controls. These results suggest that non-carrier relatives and controls have similar characteristics, and only c.-405G carriers present alterations in plasma TC, LDLc and glucose levels.

### 3.5. Oral Glucose Tolerance Test

An OGTT was performed in four carriers from the family (subjects 104.01, 104.02, 104.03 and 104.05). To generate a more specific control sample, we randomly selected 28 individuals matched for age, gender and BMI (7 per carrier). 

The results of the OGTT are shown in Table 2. Carriers had significantly higher fasting glucose levels (*p* = 0.049), glucose at 60 min (*p* = 0.002), area under the curve (AUC) of glucose (*p* = 0.027) and insulin levels at the 60 min point (*p* = 0.027). Interestingly, carriers showed lower fasting insulin levels than did the control group (in the limit of significance) and a significantly delayed insulin response after glucose intake (peak at 60 min). These results, even after having included the two youngest mutation carriers (25 and 29 years), who present the lowest glucose levels and BMI among the c.-405G carriers (subjects 104.01 and 104.02, Figure 1), Further suggest that glucose metabolism is altered in mutation carriers with higher glucose and insulin levels. 

## 4. Discussion

The role of *SREBF2* in lipid metabolism makes this gene a candidate responsible for ADH, a group of very frequent diseases that predispose one to atherosclerosis. We screened a sample of ADH patients without mutations in the genes known to cause the disease (*LDLR*, *APOB* and *PCSK9*). We found several variations in the analyzed genes. The only one expected to have a pathogenic effect was c.-405A>G, which is in the promoter region of the *SREBF2* gene. This mutation was found in one patient, named subject 104.00, and in their family.

A comparison of promoter sequences from several mammals showed that this position is conserved in the five *Hominidae* analyzed (Supplementary Figure S1), suggesting its importance in gene regulation and a possible effect if it is altered.

We tried to demonstrate the role of this mutation as being responsible for ADH and increased levels of glucose in plasma in several ways. Firstly, we screened the variation in 429 healthy controls with lipid and glucose levels within the normal range and in around 1000 subjects from the general population. None of them presented the c.-405G allele. Secondly, we studied the proband’s family, composed of 12 co-sanguineous subjects, and all patients clinically diagnosed as having ADH were heterozygous carriers for c.-405A>G. We compared their glucose levels and response to glucose overload to other family members and controls. Thirdly, we studied whether this variation could alter protein transcription by transfecting different kinds of cells with a plasmid containing the variation, and our results indicate that this mutation increases the transcription of the gene. These cells may represent different cell types (hepatocyte, intestinal endothelium and adipocyte cell types) where *SREBF2* expression is important. *SREBF2* expression levels are different in each cell type (Figure 2), and the increase in the expression levels of c.-405A>G seems to be different in each one (about 2×, 2.6× and 3.6×, respectively). Increased expressions of SREBP2 in the liver, intestinal endothelium and adipocyte produce increased levels of cholesterol in blood in animal models [26,27]. 

The cholesterol levels in family 104 were mildly hypercholesterolemic. The SREBP2 promoter variant increases SREBP2 transcription, but the exact mechanism by which hypercholesterolemia is produced is not known. 

Previous findings have suggested the possibility that *SREBF2* mutations are a cause of hypercholesterolemia [8]. *SREBF2* has also been shown to modulate cholesterol levels: the p.A595G polymorphism diminishes *SREBF2* cleavage in vitro and is associated with higher cholesterol levels in patients with polygenic hypercholesterolemia [28]. Other investigators [29] have demonstrated that SNPs in this gene may influence cholesterol levels in ADH patients who also have mutations in *LDLR*. In the present report, we show for the first time the cosegregation of an *SREBF2* rare variant with the ADH phenotype and with increased gene transcription in three cellular lines. SREBP2 is a central regulator of cholesterol synthesis, and it is known that an increase in SREBP2 activity causes an elevation in cholesterol synthesis [26,30]. It has also been shown that the activation of cholesterol synthesis leads to hypercholesterolemia [4,26,31]. The *SREBF2* c.-405A>G gene promoter variation increases transcription levels in the cells tested. Therefore, this increase in SREBP2 protein levels may facilitate its activation and, as a consequence, an elevation in cholesterol synthesis that would lead to the hypercholesterolemia presented by carriers.

Interestingly, we observed that c.-405G carriers from family 104 presented higher glucose and insulin levels, suggesting the role of this variation as a glucose metabolism modulator. Proband 104.00 was hyperglycemic, and the remaining carriers presented glucose levels characteristic of prediabetic patients. We excluded the possibility that these patients present the most common forms of MODY by sequencing *GCK*, *HNF1A* and *HNF4A*.

The OGTT response observed in carriers was similar to that found in diabetic and prediabetic patients. An increased adipose tissue mass is a key component of diabetic and prediabetic states [32]. However, the BMI in this family was not elevated (four out of seven with BMI < 23 kg/m^2^, two approximately 25 kg/m^2^ and one around 28 kg/m^2^), excluding obesity as the primary cause of hyperglycemia. It is further relevant to note that the two carriers with the lowest glucose levels had fasting values close to 100 mg/dL in association with an altered OGTT response, although both were at a low average risk for diabetes using classical parameters (BMI < 20kg/m^2^, age < 30 years) (Table 1 and Figure 1). 

Although *SREBF2* has mainly been associated with lipid metabolism, there are relevant data that can relate this gene to glucose metabolism in humans and in animals. However, no mutation that can modulate the levels of glucose has previously been found. Our findings seem to confirm the role of this gene in glucose metabolism, as previously suggested by several studies [33]. These authors worked with transgenic mice overexpressing SREBP2 in pancreatic β-cells. Isolated β-cells presented lower insulin content, and they were smaller and fewer in number. These authors showed that the activation of SREBP2 in these cells can cause diabetes by a mechanism involving the loss of β-cells and insulin secretion. Furthermore, they showed that this cell dysfunction is due to an accumulation of cholesterol within the cell. Their data in experimental animals are in agreement with our findings in humans.

In summary, we showed that a mutation in *SREBF2* that increases gene transcription may be associated with an inherited increase in plasma cholesterol and glucose levels. In order to demonstrate a causative role of this mutation in a patient’s phenotype, further studies on the expression of *SREBF2* and the genes involved in its regulation should be performed in patient cells, together with in vitro studies regarding mutant protein localization.

## Figures and Tables

**Table 1:** Comparison between carriers, non-carrier relatives and controls.

Parameters	Carrier Relatives	Non-Carrier Relatives	Controls	*p* Values Carriers vs. Controls	*p* Values Carriers vs. Non-Carriers	*p* Values Non-Carriers vs. Controls
*n*	7	5	429			
Age	50.9 ± 20.1	37.6 ± 5.7	43.9 ± 26.4	0.2678	0.1879	0.3903
BMI	22.9 ± 22.9	24.5 ± 3.3	24.9 ± 3.1	0.0993	0.4583	0.7807
Waist	84.9 ± 13.4	85.4 ± 12.2	83.9 ± 10.9	0.8189	0.9444	0.7604
Glucose	108.1 ± 8.9	88.2 ± 8.2	89.3 ± 13.9	0.0004	0.0027	0.859
TC	240 ± 30.4	173.4 ± 11.1	172.5 ± 19	0.0000	0.0009	0.9191
LDLc	164.1 ± 26.1	88.6 ± 23	93.6 ± 20.5	0.0000	0.0004	0.5892

Data are expressed as mean ± SD. Age in years; BMI in Kg/m^2^; Waist: waist circumference in cm; glucose levels and lipid parameters in mg/dL. BMI: Body Mass Index. TC: total cholesterol. LDLc: LDL cholesterol.

**Table 2:** Results of OGTT.

Parameters	Carriers	Controls	*p* Values
*n*	4	28	
Age (years)	50.8 ± 20.1	42.3 ± 10.2	0.281
BMI (Kg/m^2^)	22.9 ± 3.6	24.7 ± 2.8	0.342
Waist (cm)	84.8 ± 13.4	86.1 ± 11.4	0.856
Glucose 0 min	108.1 ± 8.9	89.3 ± 32.2	0.049 *
Glucose 30 min	164.1 ± 26.1	112.8 ± 33.2	0.107
Glucose 60 min	147.5 ± 14.1	125.6 ± 38.6	0.002 *
Glucose 90 min	124.0 ± 10.6	104.8 ± 22.9	0.049 *
Glucose 120 min	114.2 ± 26.0	88.6 ± 20.5	0.071
AUC Glucose	16,193 ± 1258	13,757 ± 2673	0.027 *
Insulin 0 min	7.1 ± 2.7	9.9 ± 3.1	0.050 *
Insulin 30 min	41.5 ± 22.8	55.1 ± 25.6	0.429
Insulin 60 min	91.5 ± 29.2	60.4 ± 19.1	0.042 *
Insulin 90 min	44.1 ± 13.3	42.1 ± 42.1	0.864
Insulin 120 min	54.8 ± 23.3	27.7 ± 14.9	0.283
AUC Insulin	6254 ± 1769	5296 ± 1615	0.316
HOMA	1.8 ± 0.8	2.1 ± 0.8	0.712

SREBP2 c.-405A>G carriers and controls matched by age, gender and BMI. A non-parametric test for two independent samples (Mann–Whitney U) was used (Min: minute of blood extraction at the OGTT; glucose and Insulin units are mg/dL and µU/mL, respectively). Data are expressed as mean ± SD (* significant; AUC: area under the curve; HOMA: Homeostasis Model Assessment).

## Data Availability

The data presented in this study are available on request from the corresponding author.

## Supplementary Materials

The following supporting information can be downloaded at: https://www.mdpi.com/article/10.3390/biomedicines10051178/s1, Figure S1: Comparison of the c.-405A>G position in SREBF2 gene in five *Hominidae* species; Table S1: Sequences of primers used in this work.
