# Supplementary material for: A Very Rare Variant in SREBF2, a Possible Cause of Hypercholesterolemia and Increased Glycemic Levels
Source: Biomedicines. 2022 May 19;10(5):1178. doi: 10.3390/biomedicines10051178 (PMC9138625; doi:10.3390/biomedicines10051178)
Supplement: Supplementary file 1 [file biomedicines-10-01178-s001.zip › biomedicines-1680071-SM.pdf]

Supplementary Material

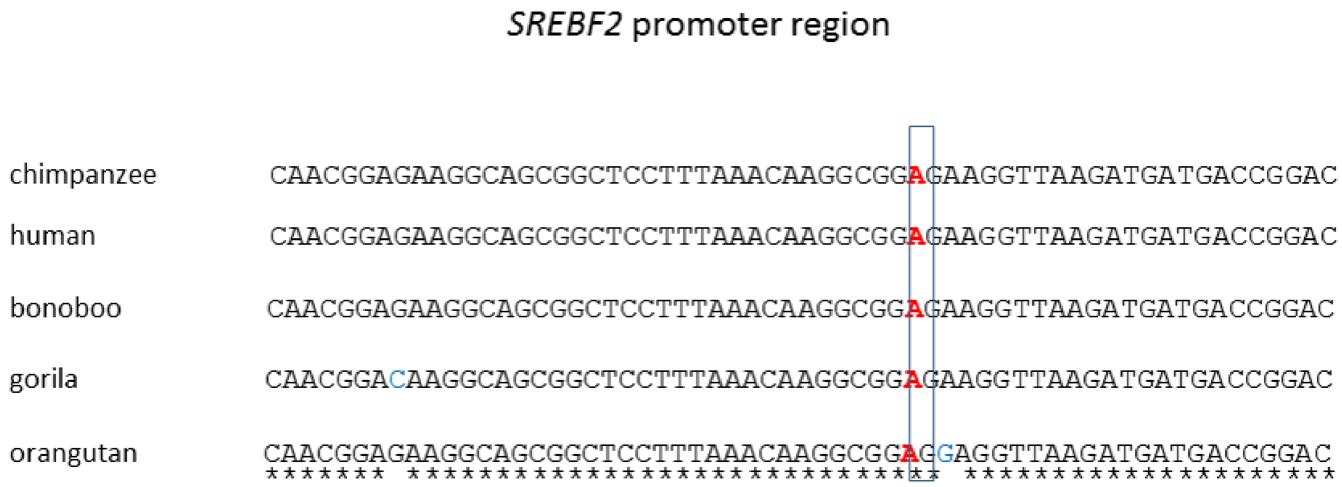

Figure S1. Comparison of the c.-405A>G position in SREBF2 gene in five *Hominidae* species.
